# Genetic polymorphisms of *GPR126* are functionally associated with PUMC classifications of adolescent idiopathic scoliosis in a Northern Han population

**DOI:** 10.1111/jcmm.13486

**Published:** 2018-01-24

**Authors:** Gang Liu, Sen Liu, Mao Lin, Xiaoxin Li, Weisheng Chen, Yuzhi Zuo, Jiaqi Liu, Yuchen Niu, Sen Zhao, Bo Long, Zhihong Wu, Nan Wu, Guixing Qiu

**Affiliations:** ^1^ Department of Orthopedic Surgery Peking Union Medical College Hospital Peking Union Medical College and Chinese Academy of Medical Sciences Beijing China; ^2^ Beijing Key Laboratory for Genetic Research of Skeletal Deformity Beijing China; ^3^ Department of Central Laboratory Peking Union Medical College Hospital Peking Union Medical College and Chinese Academy of Medical Sciences Beijing China; ^4^ Medical Research Center of Orthopedics Chinese Academy of Medical Sciences Beijing China

**Keywords:** adolescent idiopathic scoliosis, *GPR126/ADGRG6*, single nucleotide polymorphism, PUMC classification

## Abstract

*GPR126* has been identified to be associated with AIS (Adolescent Idiopathic Scoliosis) in different populations, but data on the northern Chinese population are unavailable. Additionally, it is important to know the exact clinical phenotypes associated with specific genetic polymorphisms. Fourteen SNP (single nucleotide polymorphism) loci in *GPR126* were genotyped in 480 northern Chinese Han AIS patients and 841 controls. These patients were classified into three types based on the PUMC classification system. Luciferase assays were used to investigate their regulation of *GPR126* transcription activity. Combined and stratified genotype–phenotype association analyses were conducted. The alleles rs225694, rs7774095 and rs2294773 were significantly associated with AIS (*P* = 0.021, 0.048 and 0.023, respectively). rs225694 and rs7774095 potentially have regulatory functions for the *GRP126* gene. Correlation analysis revealed that allele A of rs225694 was a risk allele only for PUMC type II AIS (*P* = 0.036) and allele G of rs2294773 was a risk allele only for PUMC type I AIS (*P* = 0.018). In summary, rs225694, rs7774095 and rs2294773 are significantly associated with disease in northern Chinese Han AIS patients. The SNPs rs225694 and rs2294773 are associated with different AIS PUMC classifications.

## Background

AIS is the most common spinal deformity, affecting approximately 1–3% of children throughout the world 1, 2. There are many classification systems, including the King classification 3, Lenke classification 4 and the Peking Union Medical College (PUMC) classification systems 5. According to the PUMC classification, AIS can be classified into single (I), double (II), or triple (III) curve types. The PUMC classification is a three‐dimensional classification system that is relatively simple to use and has corresponding recommended surgical approaches 5. Despite decades of research on AIS, its exact aetiological and pathological causes remain unclear.

Previous studies have suggested that genetic polymorphisms play a pivotal role in the pathogenesis of AIS 6, 7. Although a recent genomewide association study (GWAS) and other studies have identified several susceptible gene variants, such as *GPR126* 8, *LBX1* 9, *PAX1* 10, *BCN2* 11 and *PTK7* 12, few of these variants have been replicated in different populations, and even fewer have been analysed with regard to different AIS subtypes.

*GPR126* (encoding G protein‐coupled receptor 126), also known as *ADGRG6*, located at 6q24.1, is 144,348 bp in length and consists of 25 exons. According to Monk *et al*. 13, g*pr126* is essential for mouse survival due to Schwann cell myelination. Courtney *et al*. also found 14 that a *gpr126* deletion in cartilage could lead to idiopathic scoliosis and pectus excavatum in mice. It has been shown that *GPR126* gene SNPs are associated with AIS. Kou *et al*. 8 identified rs6570507 as a susceptibility locus with AIS in 1,819 Japanese cases and 25,939 controls and found that the developing spine of a *gpr126* knockdown zebrafish model had delayed ossification. Subsequently, Xu *et al*. 15 found this locus and two other loci (rs7774095 and rs7755109) in 352 southern Chinese cases and 149 controls. Recently, Qin *et al*. 16 found another functional locus (rs9403380) that regulates *GPR126* expression in the paraspinal muscles of southern Chinese AIS patients.

As far as we know, there has not been an association analysis of the *GPR126* gene with AIS in the northern Chinese Han population. It will be of great significance to identify the specific subgroups of AIS that are associated with *GPR126* genetic polymorphisms. We suggested that *GPR126* risk SNPs are potentially associated with the AIS PUMC classification system. Therefore, we enrolled a cohort from the northern Chinese Han population and corresponding controls. SNPs in *GRP126* were genotyped with subsequent phenotypic association analysis based on their PUMC classification.

## Materials and methods

### Participants

A total of 480 patients diagnosed with AIS and 841 healthy in‐house controls from a northern Chinese Han population at Peking Union Medical College Hospital were enrolled between July 2011 and August 2016. The inclusion and exclusion criteria were as follows:

Inclusion criteria

1. Diagnosed as AIS with a Cobb angle > 20 degrees.
2. Age of onset between 10 and 18 years.
3. Origin was from the northern Chinese Han population as identified by a Native Place questionnaire.
4. Having complete imaging data, including X‐ray, three‐dimensional imaging of the spine CT or spinal MRI.

Exclusion criteria

1. Scoliosis secondary to a known aetiology, including congenital scoliosis, neuromuscular scoliosis, scoliosis secondary to skeletal dysplasia or connective tissue abnormalities.
2. Origin was not from the northern Chinese Han population.
3. Incomplete imaging data.
4. Having a chronic disease that influenced skeletal development.

All patients diagnosed as AIS were classified into three types based on the PUMC classification system 5 by at least two experienced orthopaedic surgeons. Written informed consent was obtained from all participants or their parents. The Ethics Committee of Peking Union Medical College Hospital, Chinese Academy of Medical Sciences approved this study.

### SNP selection and genotyping

Fourteen candidate SNPs around *GPR126* were selected by the following criteria from a literature review and the NCBI SNP database (www.ncbi.nlm.nih.gov/SNP; Table S1): (*i*) SNP with a minor allele frequency (MAF) above 5%; (*ii*) Tag SNPs were preferred; (*iii*) reported SNPs associated with idiopathic scoliosis; and (*iv*) potential functional SNPs predicted by HaploReg (v4.1) 17.

Genomic DNA was extracted from the peripheral blood of each participant using DNeasy Blood & Tissue Kits (QIAGEN, Eastwin Scientific, Inc. Beijing, China). SNP genotyping was performed using the MassArray system from Sequenom. All of the details were the same as in our previous studies 18, 19, 20.

### Luciferase assay

We used luciferase assays to test the influence of significantly associated SNPs on transcription. We cloned oligonucleotides around each SNP (Table S2) and cloned them into a pGL3 promoter luciferase reporter vector (Promega, Madison, WI, USA). Human embryonic kidney 293a cells (HEK293a) and HeLa cells were transfected with the reporter vector by lip3000 (Invitrogen, Carlsbad, CA, USA). After a 48‐hr incubation, cells were harvested and luciferase activity was measured using a Dual‐Luciferase Reporter Assay System (Promega, Madison, WI, USA).

### Data analysis

Hardy–Weinberg equilibrium (HWE) tests and primary analyses, including allelic, genotypic, haplotypic analyses and linear regression controlling for sex were conducted using PLINK software (v1.07, 2009 Shaun Purcell) and the Haploview program (version 4.2, Broad Institute of MIT and Harvard, Cambridge, MA, USA). The baseline characteristics of AIS participants presenting different PUMC types were compared through SPSS software (16.0 version, SPSS Inc., Chicago, IL, USA). Intergroup differences were assessed with the chi‐square test for categorical variables and one‐way anova for continuous normally distributed variables.

## Results

### Baseline characteristics of participants

Seventy‐eight males and 402 females were enrolled in the case group, and 483 males and 358 females were enrolled in the control group (Table 1). All patients included in our study were classified into three different PUMC AIS types based on their radiology data (Fig. S1). There were no significant differences in the main Cobb angle, age or gender between each of the PUMC groups (Table 1).

**Table 1:** Characteristics of three PUMC types of AIS patients

Variables	Cases^a^				Control
	PUMC type I	PUMC type II	PUMC type III	In total	
Number	146	273	61	480	841
Gender M:F	27:119	40:233	11:50	78:402	483:358
Mean age	13.57 ± 1.93	13.37 ± 1.96	13.59 ± 1.93	13.46 ± 1.94	NA
Main Cobb angle	49.22 ± 16.95	50.55 ± 14.16	53.23 ± 14.53	50.49 ± 15.12	NA

a *P* value of Gender/Mean age/main Cobb angel was greater than 0.05 within groups; NA, not available.

### Genotyping and Hardy–Weinberg equilibrium test

Among all participants, 14 SNPs in or around *GPR126* were genotyped. rs6929442 was excluded from subsequent analyses because of the low call rate (Table S1). The other 13 SNPs were successfully genotyped with a minimum call rate of 98%. None of the 13 SNPs deviated from HWE and, thus, were subjected to subsequent analyses 21.

### Association analysis

**Figure 1:**
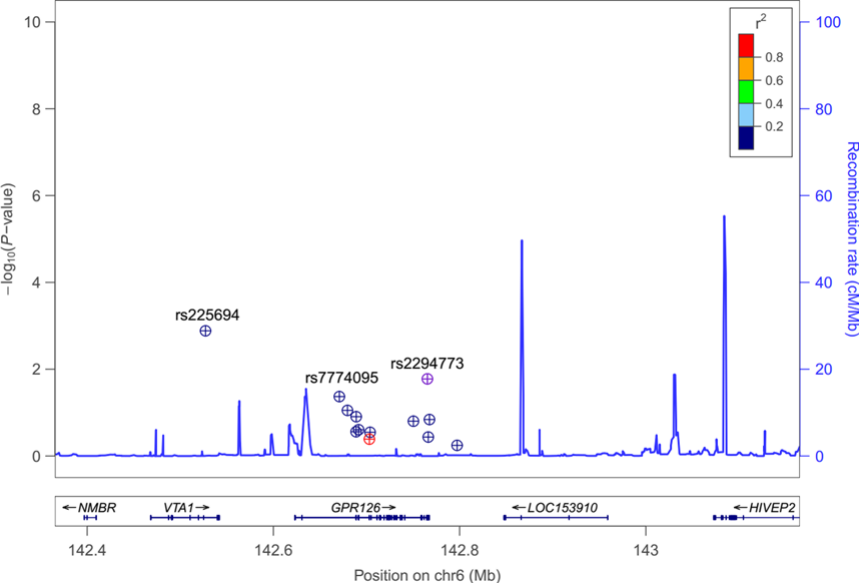
Regional Association Plot for the *GPR126* candidate loci on 6q24. The ‐log_10_ (*P* value) of the candidate SNP loci association with adolescent idiopathic scoliosis was plotted with LocusZoom software. The SNPs with the highest association signal were rs225694, rs2294773 and rs7774095.

Of the 13 SNPs, rs225694, rs7774095 and rs2294773 presented significant allelic differences between the case and control groups. This result was demonstrated with LocusZoom, which showed that the alleles of rs225694, rs2294773 and rs7774095 had the highest association signal with AIS (http://csg.sph.umich.edu/locuszoom/) (Fig. 1). Controlling for sex, allele A of rs225694, allele A of rs774095 and allele G of rs2294773 had risk effects based on the different test models (*P* = 0.021, 0.048 and 0.023, respectively; Table 2).

**Table 2:** Association results of tested SNPs with AIS whilst controlling for sex

SNP	Allele (1/2)	Test	OR	*P* *
rs225694	A/G	ADD	0.8749	0.8378
		DOMDEV	2.409	0.211
		GENO 2DF	NA	**0.0208**
rs7774095	A/C	ADD	1.224	**0.0481**
		DOMDEV	1.02	0.885
		GENO 2DF	NA	0.08038
rs6570507	A/G	ADD	1.187	0.08712
		DOMDEV	1.01	0.9426
		GENO 2DF	NA	0.169
rs35699755	T/C	ADD	1.563	0.4273
		DOMDEV	0.7156	0.5784
		GENO 2DF	NA	0.6481
rs17280293	G/A	ADD	NA	NA
		DOMDEV	NA	NA
		GENO 2DF	NA	NA
rs11155242	C/A	ADD	0.9789	0.9444
		DOMDEV	0.8723	0.6919
		GENO 2DF	NA	0.6573
rs2143390	T/C	ADD	1.241	0.1668
		DOMDEV	0.888	0.5333
		GENO 2DF	NA	0.3362
rs4896582	G/A	ADD	0.8017	0.1006
		DOMDEV	1.175	0.3417
		GENO 2DF	NA	0.2575
rs7755109	G/A	ADD	1.121	0.2292
		DOMDEV	1.092	0.5039
		GENO 2DF	NA	0.2732
rs2294773	G/C	ADD	1.191	0.2659
		DOMDEV	1.193	0.348
		GENO 2DF	NA	**0.0233**
rs2294775	G/C	ADD	0.8286	0.5173
		DOMDEV	1.109	0.7514
		GENO 2DF	NA	0.7197
rs3748069	G/A	ADD	1.134	0.1949
		DOMDEV	1.087	0.5332
		GENO 2DF	NA	0.224
rs7763064	A/G	ADD	1.126	0.2386
		DOMDEV	0.7834	0.08444
		GENO 2DF	NA	0.1994

ADD, additive effects of allele dosage test. DOMDEV tests a variable coded 0,1,0 for the three genotypes A1A1, A1A2, A2A2. GENO 2DF tests the coefficients for ADD and DOMDEV together. OR, odds ratio. NA, not available. *Chi‐square test, *P*‐values for each line reflect the effect of the entity under the TEST column. The bold values mean “statistically significantly different”.

### Haplotypic analysis

We constructed haplotypes based on genotype data from thirteen SNPs using Haploview software (version 4.2). The pairwise linkage disequilibrium r‐square values between SNPs and the LD plots are presented in Figure S2. According to the Four Gamete Rule, we identified two haplotype blocks: rs7774095 and rs6570507 were in one block and rs35699755, rs17280293, rs11155242, rs2143390, rs4896582, rs7755109, rs2294773, rs2294775 and rs3748069 were in another block. There was no significant association between these two haplotype blocks and AIS when controlling for sex (Table 3).

**Table 3:** Conditional haplotype‐based association testing in *GPR126* gene with AIS whilst controlling for sex

	Haplotype	Frequency	OR	*P* Value
Block 1	AA	0.337	(‐ref‐)	0.0668
	CA	0.0159	0.6605	
	CG	0.646	0.8171	
Block 2	TAACAGCCG	0.0397	(‐ref‐)	0.0134
	CAATAGGCG	0.203	0.85	
	CAACAACCA	0.365	0.7271	
	CAACGACCA	0.218	0.6555	
	CGCCAGCGG	0.0194	0.5834	
	CACCAGCGG	0.0522	0.6356	
	CAACAGCCA	0.0221	0.5847	
	CAACAGGCG	0.0143	2.666	
	CAACGGCGG	0.0101	0.8706	

Block 1, rs7774095‐rs6570507, three common haplotypes (MHF, minimum haplotype frequency ≥0.01) from four possible. Block 2, rs35699755‐rs17280293‐rs11155242‐rs2143390‐rs4896582‐rs7755109‐rs2294773‐rs2294775‐rs3748069, nine common haplotypes (MHF ≥ 0.01) from 37 possible (‐ref‐), haplotype been selected to be the baseline, reference category. Unadjusted *P*‐value < Bonferroni‐corrected *P*‐value was considered statistically significant.

### Luciferase assay

**Figure 2:**
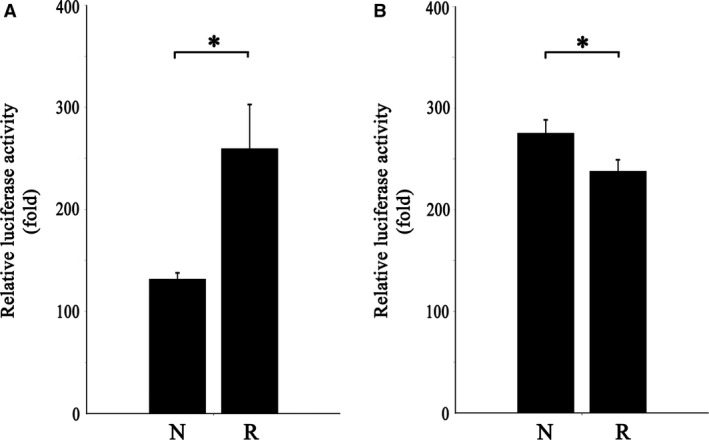
Relative Luciferase Activity for different allele of rs225694 and rs7774095. (**A**) Transcriptional enhancer activities of rs225694 constructs. The construct containing the risk allele (R) had 1.97‐fold higher level relative luciferase activity than the non‐risk allele. *P value < 0.05. Error bars indicate stand error. The assay was repeated three times. (**B**) Transcriptional enhancer activities of rs7774095 constructs. The construct containing the risk allele (R) had 0.86‐fold lower level of relative luciferase activity than the non‐risk allele. *P value < 0.05. Error bars indicate stand error. The assay was repeated three times.

Among the three SNPs with positive results in association analyses, rs225694 and rs7774095 had potential enhancer activity. The luciferase activity of the risk allele and no‐risk allele was compared. The construct containing risk allele A of rs225694 had a 1.97‐fold higher level of luciferase activity than non‐risk allele C (*P* = 0.007). The construct containing risk allele A of rs7774095 had a 0.86‐fold lower level of luciferase activity than the non‐risk allele C (*P* = 0.015; shown in Fig. 2). Experiments using the HeLa cell line showed similar results (Fig. S3).

### PUMC subgroup analysis

We conducted PUMC subgroup analysis in the three SNPs with positive association results. The analysis indicated that the genotypic association of rs225694 had positive results for the PUMC type II subgroups (*P* = 0.036). rs2294773 presented a positive result for the PUMC type I group (*P* = 0.018), and the other subgroups showed no significant difference (Table 4).

**Table 4:** Peking Union Medical College (PUMC) subgroup analysis of three associated SNPs whilst controlling for sex

SNP	Allele (1/2)	Test model	ALL		PUMC type I		PUMC type II		PUMC type III	
			OR	*P*	OR	*P*	OR	*P*	OR	*P*
rs225694	A/G	ADD	0.8749	0.8378	6.10E‐05	0.9991	4.52E‐05	0.999	2.456	0.168
		DOMDEV	2.409	0.211	3.50E+04	0.999	4.99E+04	0.9989	0.8687	0.8636
		GENO 2DF	NA	**0.0208**	NA	0.1246	NA	**0.03641**	NA	0.1399
rs7774095	A/C	ADD	1.224	**0.0481**	1.323	0.05793	1.213	0.1166	1.038	0.8686
		DOMDEV	1.02	0.885	1.014	0.9441	1.06	0.7272	0.8856	0.6925
		GENO 2DF	NA	0.08038	NA	0.1199	NA	0.1598	NA	0.9247
rs2294773	G/C	ADD	1.191	0.2659	1.332	0.1859	1.019	0.9233	1.272	0.3996
		DOMDEV	1.193	0.348	1.271	0.3587	1.379	0.1696	0.7072	0.359
		GENO 2DF	NA	**0.0233**	NA	**0.01841**	NA	0.09374	NA	0.6177

ADD, additive effects of allele dosage test. DOMDEV tests a variable coded 0,1,0 for the three genotypes A1A1, A1A2, A2A2. GENO 2DF tests the coefficients for ADD and DOMDEV together. OR, odds ratio. NA, not available. *Chi‐square test, *P*‐values for each line reflect the effect of the entity under the TEST column. The bold values mean “statistically significantly different”.

## Discussion

In this study, we investigated the association of fourteen SNP loci in the *GPR126* gene with AIS in a northern Chinese Han population. We found a significant association signal of rs7774095 with AIS. Moreover, we identified two novel SNP loci (rs225694 and rs2294773) that not only contributed to predisposition but were also associated with different types of AIS.

**Figure 3:**
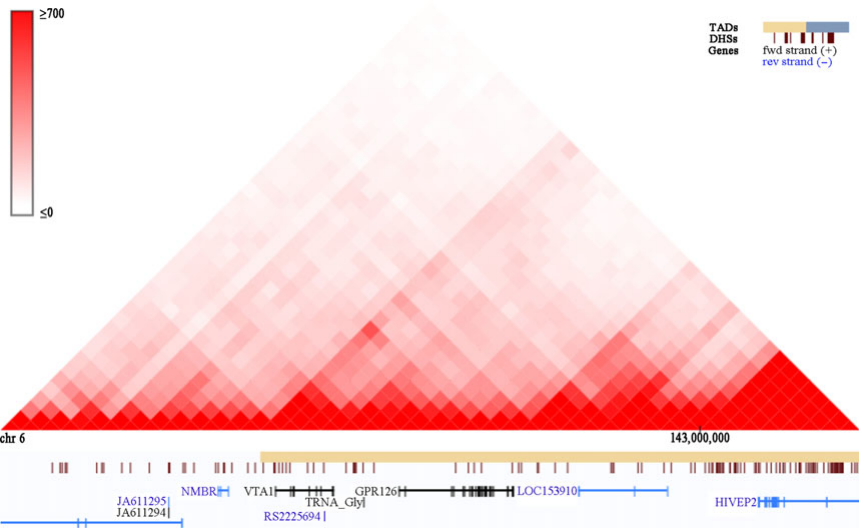
Topologically associated domain (TAD) of *GPR126* and *VTA1*. Topologically associated domains were plotted by Hi‐C website. Genes *GPR126* and *VTA1* were in the same topologically associated domain.

rs225694 was first identified to be associated with height 22. It is located in an intronic area of *VTA1*, but is in the same topologically associated domain (TAD) as *GPR126* (Fig. 3). TAD is the compartment of the 3D chromatin space in which genomes of many bilaterian animals are organized. It is universally recognized that TAD plays an important role in gene regulation 23 and has potential gene‐enhancer interactions 24. Thus, rs225694 is supposed to have enhancer activity for the gene *GPR126*. Association analysis showed that this allele was most significantly associated with AIS, controlling for sex. Luciferase assays confirmed its enhancer function, including up‐regulation of transcription activity. Considering the PUMC classification, rs225694 had a powerful association only with type II AIS. We therefore suggested that the risk allele of rs225694 up‐regulated transcription of GPR126 and increased the risk of PUMC type II AIS.

rs7774095, an intronic SNP of *GPR126*, was first reported to be associated with AIS in a Japanese population in 2013 8. Other studies in southern Chinese populations replicated the association of this locus 15, 16. However, there was no clear functional evidence provided for this conclusion. We also observed this association and showed that it down‐regulates transcription activity. It has been shown that deletion of *gpr126* in cartilage leads to an idiopathic scoliosis deformity in a mouse model 14. However, detailed clinical phenotype analyses found no significant association with any PUMC AIS subgroup, which may be due to the limited sample size. Additional studies with larger sample sizes are needed to identify the relationship between rs7774095 and PUMC AIS subgroups.

Association analyses of rs2294773 also showed significant results with AIS. rs2294773 is located in the 3′UTR of *GPR126*. It is postulated that the 3′UTR is a regulatory region that has potential activity in regulating mRNA localization, translation and stability 25. Different alleles at one locus can allow or eliminate miRNA binding. Song *et al*. found that mRNA containing the susceptibility allele rs4148941 had an enhanced interaction with miR‐513a‐5p, which may contribute to the aetiology of Lumbar disc degeneration (LDD) 26. Zhu *et al*. found a functional SNP in the 3′UTR region associated with bone mineral density (BMD) 27. As for rs2294773, *in silico* predictions by three online tools, including TargetScan (http://www.targetscan.org/) 25, 28, 29, miRanda (http://www.microrna.org/) 30 and mirSNP (http://cmbi.bjmu.edu.cn/mirSNP) 31, showed that there are three microRNAs, hsa‐miR‐1343, hsa‐miR‐198 and hsa‐miR‐30b‐3p that can bind to this mRNA. We also performed eQTL analysis in different tissues from the database and found that rs2294773 was associated with *GPR126* gene expression in several tissues, including transformed fibroblasts, sun‐exposed skin and so on (Table S3, Fig. S4). Although there was no eQTL data for cartilage or bone, this locus may influence *GRP126* gene expression and potentially contribute to AIS formation by these above miRNAs. Regarding the PUMC classification, rs2294773 also had a powerful association with only type I AIS. Additional replication studies with larger sample sizes are needed to provide further evidence for this association.

In our study, we identified two haplotype blocks in the candidate *GPR126* gene with AIS. In the first block, we found that the A‐A haplotype of rs7774095‐rs6570507 was not significantly associated with AIS (*P* = 0.067), although previous studies had identified an association of rs6570507 with AIS. In contrast to previous studies, the lack of evidence of an association between rs6570507 and AIS may be due to different allele frequencies between geographic populations or the limitation of the sample size studied 8, 15, 32. In the second block, we also did not find significant association.

In conclusion, our study found that three SNPs near or in *GPR126* were associated with AIS in the northern Chinese Han population. Risk allele A of rs225694 had higher transcription activity, and risk allele A of rs7774095 had lower transcription activity *in vitro*. We suggested that both overexpression and underexpression of *GPR126* increased AIS susceptibility for different types of AIS and that SNPs at microRNA binding sites potentially contribute to AIS. *GPR126* risk SNPs had potential associations with the type of AIS, as defined by the PUMC classification system. Rs225694 was functionally associated with PUMC type II AIS, and rs2294773 was mainly functionally associated with type I AIS, which contribute to our understanding of AIS development. SNPs of *GRP126* were phenotypically associated with distinct PUMC types of AIS and might be useful as potential biomarkers for AIS classification and further surgical intervention.

## Conflict of interest

The authors had no conflict of interest.

## Supporting information


**Figure S1 X‐ray image of AIS patients with PUMC classification system**. A/B/C: adolescent idiopathic scoliosis with PUMC type I/II/III.

**Figure S2 Linkage Disequilibrium (LD) structures of the thirteen candidate SNPs genotyped in *GPR126* gene**. The numbers inside the diamonds indicate the r‐square value for pairwise analysis. The LD strength between paired SNPs are shown in color of the diamonds according to the confidence interval's model.

**Figure S3 Relative Luciferase Activity for different allele of rs225694 and rs7774095 in HeLa cell line**. (A) Transcriptional enhancer activities of rs225694 constructs. The construct containing risk‐allele (R) had 1.97‐fold higher level of relative luciferase activity than non‐risk allele. *P value <0.05. Error bar, stand error. The assay was repeated three times. (B) Transcriptional enhancer activities of rs7774095 constructs. The construct containing risk‐allele (R) had 0.86‐fold lower level of relative luciferase activity than non‐risk allele. *P value <0.05. Error bar, stand error. The assay was repeated three times.

**Figure S4 EQTL box plot of rs2294773 in different tissues**.

**Table S1.** Fourteen target SNPs of *GRP126* from literature review.
**Table S2.** Oligonucleotide sequence for luciferase assay.
**Table S3.** EQTL analysis result of rs2294773 in different tissues.
