# Supplementary figures and images for: Genetic polymorphisms of GPR126 are functionally associated with PUMC classifications of adolescent idiopathic scoliosis in a Northern Han population
Source: J Cell Mol Med. 2018 Jan 24;22(3):1964–71. doi: 10.1111/jcmm.13486 (PMC5824397; doi:10.1111/jcmm.13486)

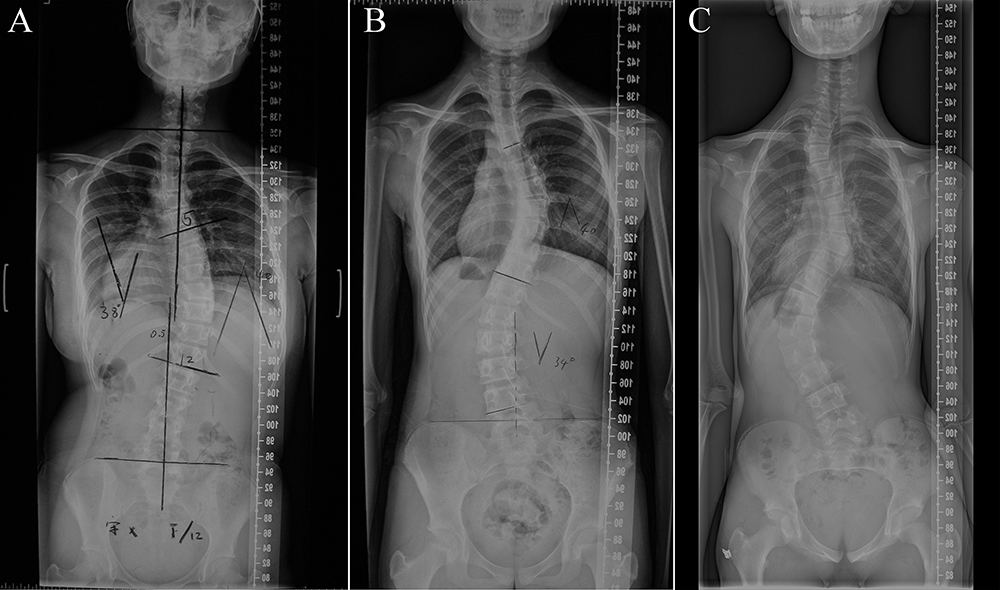

Supplement: Supplementary file 1 — Figure S1 X‐ray image of AIS patients with PUMC classification system. A/B/C: adolescent idiopathic scoliosis with PUMC type I/II/III. [file JCMM-22-1964-s001.tiff]

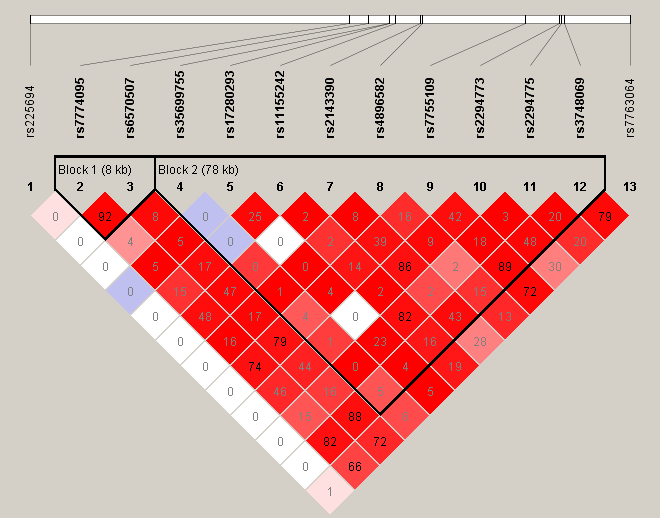

Supplement: Supplementary file 2 — Figure S2 Linkage Disequilibrium (LD) structures of the thirteen candidate SNPs genotyped in GPR126 gene. The numbers inside the diamonds indicate the r‐square value for pairwise analysis. The LD strength between paired SNPs are shown in color of the diamonds according to the confidence interval's model. [file JCMM-22-1964-s002.tiff]

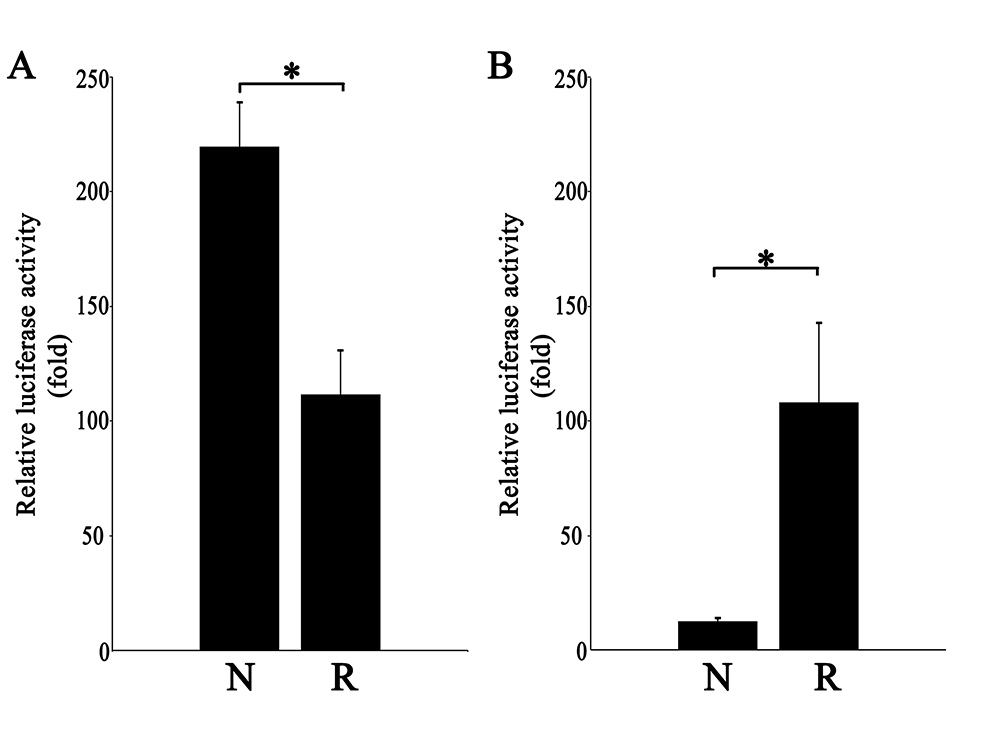

Supplement: Supplementary file 3 — Figure S3 Relative Luciferase Activity for different allele of rs225694 and rs7774095 in HeLa cell line. (A) Transcriptional enhancer activities of rs225694 constructs. The construct containing risk‐allele (R) had 1.97‐fold higher level of relative luciferase activity than non‐risk allele. *P value <0.05. Error bar, stand error. The assay was repeated three times. (B) Transcriptional enhancer activities of rs7774095 constructs. The construct containing risk‐allele (R) had 0.86‐fold lower level of relative luciferase activity than non‐risk allele. *P value <0.05. Error bar, stand error. The assay was repeated three times. [file JCMM-22-1964-s003.tiff]

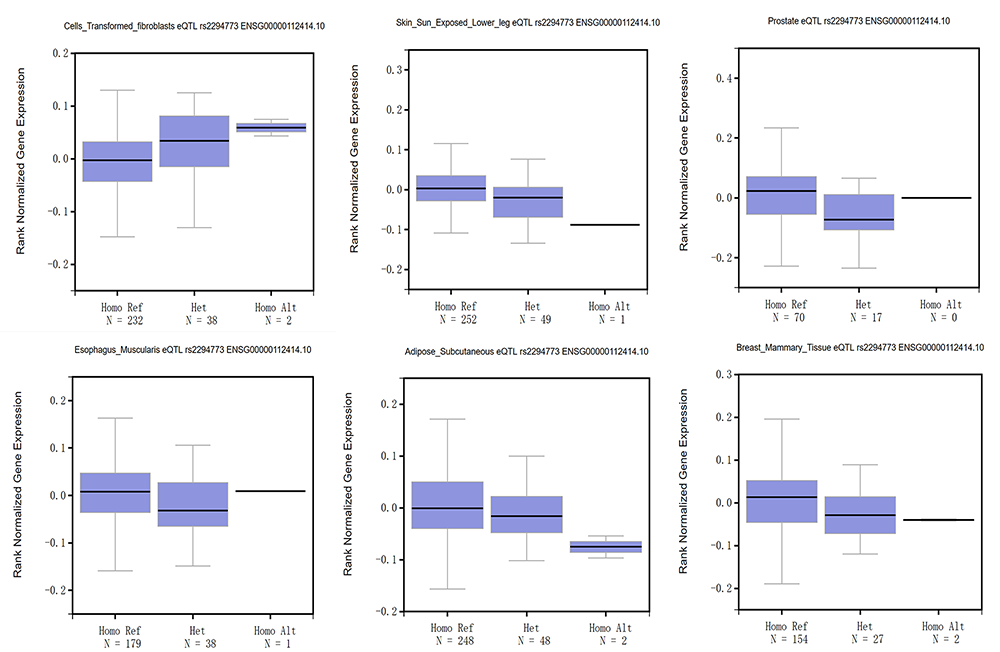

Supplement: Supplementary file 4 — Figure S4 EQTL box plot of rs2294773 in different tissues. [file JCMM-22-1964-s004.tiff]
