# Supplementary material for: Genetic polymorphisms of GPR126 are functionally associated with PUMC classifications of adolescent idiopathic scoliosis in a Northern Han population
Source: J Cell Mol Med. 2018 Jan 24;22(3):1964–71. doi: 10.1111/jcmm.13486 (PMC5824397; doi:10.1111/jcmm.13486)
Supplement: Supplementary file 5 — Table S1. Fourteen target SNPs of GRP126 from literature review. Table S2. Oligonucleotide sequence for luciferase assay. Table S3. EQTL analysis result of rs2294773 in different tissues. [file JCMM-22-1964-s005.docx]

**Table S1.** Fourteen target SNPs of *GRP126* from literature review

| **SNP ID** | **Call rate** | **BP** | **Gene symbol** | **dbSNP func annot** |
| --- | --- | --- | --- | --- |
| Rs225694 | 98% | 142527142 | VTA1/GPR126 | intron/upstream |
| Rs7774095 | 99% | 142670862 | GPR126 | intron |
| Rs6570507 | 100% | 142679572 | GPR126 | intron |
| Rs35699755 | 98% | 142688827 | GPR126 | synonymous |
| Rs17280293 | 99% | 142688969 | GPR126 | missense |
| Rs11155242 | 99% | 142691549 | GPR126 | missense |
| Rs2143390 | 98% | 142703137 | GPR126 | synonymous |
| Rs4896582 | 99% | 142703877 | GPR126 | intron |
| Rs6929442 | 90% | 142742659 | GPR126 | intron |
| Rs7755109 | 99% | 142750392 | GPR126 | intron |
| Rs2294773 | 99% | 142765405 | GPR126 | 3'UTR |
| Rs2294775 | 99% | 142766347 | GPR126 | 3'UTR |
| Rs3748069 | 99% | 142767633 | GPR126 | downstream |
| Rs7763064 | 99% | 142797289 | GPR126 | downstream |

**Table S2.** Oligonucleotide sequence for luciferase assay

| **db SNP ID** | **Sequence^*^** | |
| --- | --- | --- |
|  | **Forward** | **Reverse** |
| rs225694 | cAGTACTTCACTCAG(A/G)ATGAAATATAAATAc | tcgagTATTTATATTTCAT(T/C)CTGAGTGAAGTACTggtac |
| rs7774095 | cAGAGTAAACACACA(A/C)AAAAAAAGGTGCTGc | tcgagCAGCACCTTTTTTT(T/G)TGTGTGTTTACTCTggtac |

* Base-pair in bracket represents SNP (Risk allele/Non-risk allele)

**Table S3.** EQTL analysis result of rs2294773 in different tissues.

| **Tissue** | **P-Value** | **Effect Size** | **T-Statistic** | **Standard Error** | |  |
| --- | --- | --- | --- | --- | --- | --- |
| Cells - Transformed fibroblasts | 0.00026 | 0.38 | 3.7 | | 0.1 | |
| Skin - Sun Exposed (Lower leg) | 0.0012 | -0.24 | -3.3 | | 0.073 | |
| Prostate | 0.0022 | -0.53 | -3.2 | | 0.17 | |
| Esophagus - Muscularis | 0.017 | -0.3 | -2.4 | | 0.12 | |
| Adipose - Subcutaneous | 0.046 | -0.21 | -2 | | 0.11 | |
| Breast - Mammary Tissue | 0.048 | -0.26 | -2 | | 0.13 | |

**Figure S1. X-ray image of AIS patients with PUMC classification system.**

A/B/C: adolescent idiopathic scoliosis with PUMC type I/II/III.

**Figure S2. Linkage Disequilibrium (LD) structures of the thirteen candidate SNPs genotyped in *GPR126* gene.**

The numbers inside the diamonds indicate the r-square value for pairwise analysis. The LD strength between paired SNPs are shown in color of the diamonds according to the confidence interval’s model. .

**Figure S3. Relative Luciferase Activity for different allele of rs225694 and rs7774095 in HeLa cell line.**

(A) Transcriptional enhancer activities of rs225694 constructs. The construct containing risk-allele (R) had 1.97-fold higher level of relative luciferase activity than non-risk allele. *p value <0.05. Error bar, stand error. The assay was repeated three times.

(B) Transcriptional enhancer activities of rs7774095 constructs. The construct containing risk-allele (R) had 0.86-fold lower level of relative luciferase activity than non-risk allele. *p value <0.05. Error bar, stand error. The assay was repeated three times.

**Figure S4. EQTL box plot of rs2294773 in different tissues.**
